# Association between systemic inflammation and risk of atrial fibrillation in cancer survivors: a population-based cohort study using UK biobank

**DOI:** 10.1186/s40959-025-00414-6

**Published:** 2025-12-12

**Authors:** Quan Yang, Jiazhen Zheng, Chunting Zhao, Min Wu, Run Wang, Mingya Liu, Kai-Hang Yiu

**Affiliations:** 1 Division of Cardiovascular Medicine，Cardiac and Vascular Center, The University of Hong Kong-Shenzhen Hospital, Shenzhen, China; 2 Shenzhen Clinical Research Center for Rare Diseases, Shenzhen, 518053 China; 3 Bioscience and Biomedical Engineering Thrust, Systems Hub, The Hong Kong University of Science and Technology (Guangzhou), Guangzhou, Guangdong China; 4 Cardiology Division, Department of Medicine, The University of Hong Kong, Queen Mary Hospital, Room 1929 C, Block K, Hong Kong, China

**Keywords:** Cancer, Atrial fibrillation, Inflammation, C-reactive protein, Cardio-oncology

## Abstract

**Background:**

Cancer survivors (CSs) are at increased risk of atrial fibrillation (AF), potentially due to cancer-related inflammation and treatment effects. While inflammation has been implicated in both cancer and AF, the association between C-reactive protein (CRP) and AF risk in CSs remains unclear.

**Methods:**

We analyzed data from 19,677 UK Biobank participants (mean age 60; 34.2% male) with a prior cancer diagnosis. Incident AF was evaluated using competing-risk Cox proportional hazards models, adjusting for sociodemographic, lifestyle, and clinical factors.

**Results:**

Over a median follow-up of 10.4 years, 836 CSs (4.2%) developed AF.

Competing risk analysis revealed that the significant association between elevated CRP (> 2 mg/L) and AF risk in CSs, observed in models adjusted for sociodemographic and clinical factors (HR 1.21, 95% CI 1.06–1.37; *P* = 0.005), progressively attenuated with further adjustment for lifestyle factors (HR 1.14, 95% CI 0.99–1.31; *P* = 0.076). Despite losing statistical significance in the fully adjusted model, a consistent, suggestive trend was observed. This association was particularly pronounced in individuals not receiving radiotherapy.

**Conclusions:**

Our findings suggest that systemic inflammation is associated with an increased risk of AF among CSs, particularly in individuals without a history of radiotherapy. Further studies are needed to explore underlying mechanisms and therapeutic implications.

**Supplementary Information:**

The online version contains supplementary material available at 10.1186/s40959-025-00414-6.

## Introduction

The landscape of cancer treatment has evolved, resulting in more individuals surviving cancer diagnoses [1–3]. However, this progress has brought to light the heightened cardiovascular risks faced by cancer survivors(CSs), attributed to cancer-related inflammation and combined treatments [4–6]. Surprisingly, the risk of cardiovascular mortality in these survivors surpasses that of cancer itself [7, 8]. Atrial fibrillation (AF) emerges as a prevalent cardiovascular condition affecting 33 million individuals worldwide, with a prevalence ranging from 2 to 4% and projected to rise in the coming years [9, 10]. Notably, about 20% of cancer patients experience AF as a comorbidity [11], underscoring the substantial disease burden. The field of cardio-oncology aims to prevent and manage cancer therapy-related cardiovascular toxicity(CTR-CVT), emphasizing the integration of cancer care with cardiovascular health outcomes [12]. Nevertheless, the lack of easily identifiable and reproducible biomarkers to identify high-risk populations before AF onset poses a significant challenge [11]. 

Various studies have demonstrated inflammation as a common thread between cancer and AF, correlating with the development of both conditions [4, 13–16]. The higher prevalence of AF among cancer patients can be attributed to systemic inflammation triggered by malignancy, fostering atrial structural changes that pave the way for AF [14, 15]. This notion finds support in the elevated levels of inflammatory markers observed in cancer patients, including C-reactive protein (CRP), tumor necrosis factor α (TNF-α), and interleukins 2, 6, and 8[14], all linked to increased arrhythmic susceptibility. Despite these insights, no study has specifically examined the association between CRP and new-onset AF in CSs.

## Methods

### Study population

The research was conducted within the UK Biobank, encompassing over 500,000 individuals aged between 40 and 69 from diverse regions of the UK. Initially, detailed information on participant demographics, lifestyle factors, health history, physical measurements, and blood samples was collected. Health outcomes were linked to Hospital Episode Statistics (HES) and mortality records, categorized according to standardised International Classification of Diseases (ICD) codes.

**Fig. 1:**
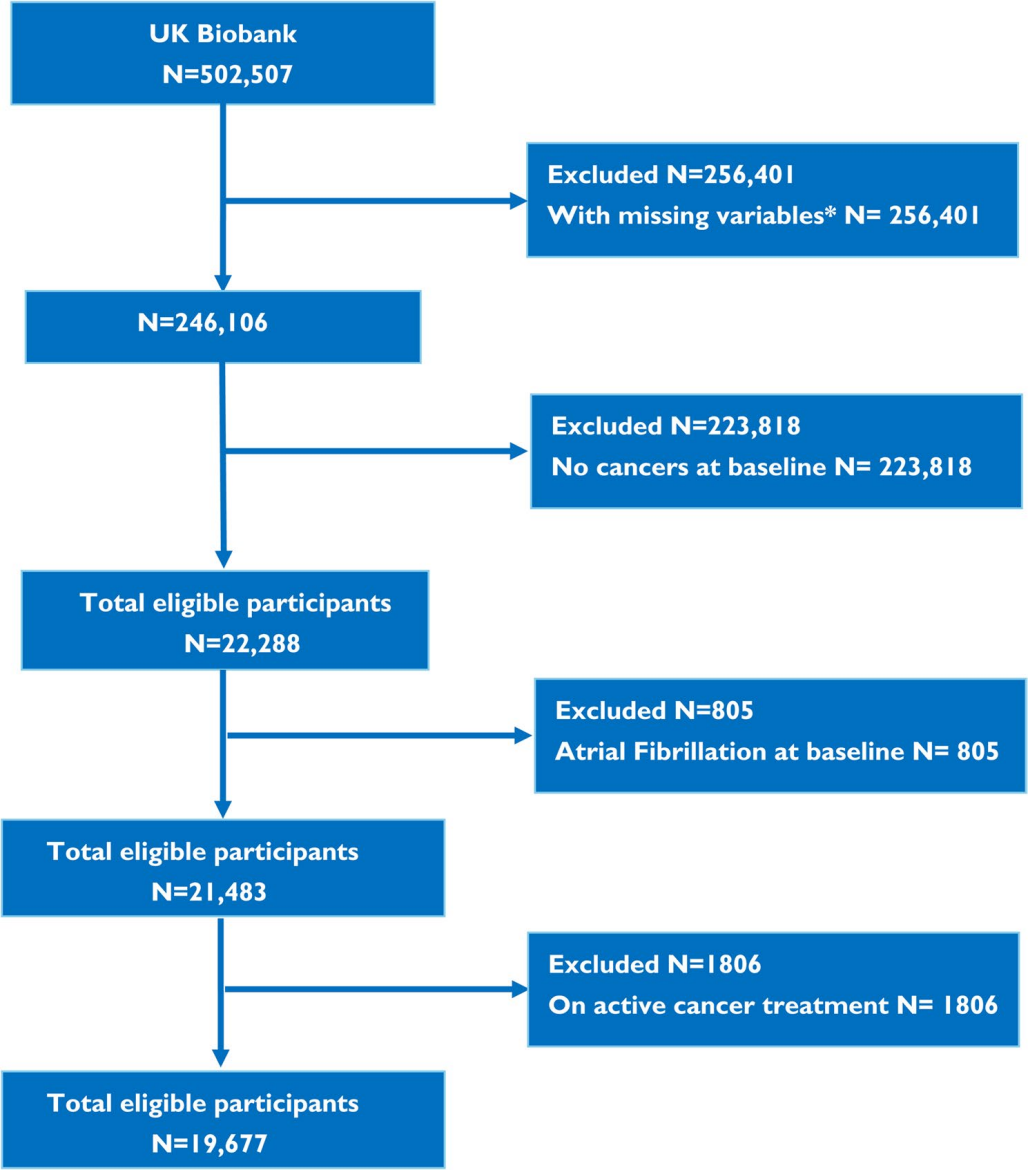
Flow diagram of the study population. *Missing variables include age, gender, race, education, body mass index, waist circumference, number of times per week consuming processed meats, number of times per day consuming fruit or vegetables, smoking history, C-reactive protein, antihypertensive medication, diabetes medication and statins

Among the UK Biobank participants, 41,867 individuals had a prior history of cancer. Those with incomplete data, study withdrawal, concurrent AF at the beginning and who received radiotherapy or chemotherapy after enrolment were excluded, resulting in a final group of 19,677 CSs (Fig. 1). The UK Biobank study received approval from the North West Multicentre Research Ethics Committee. All participants provided informed consent during their initial visit to the assessment center. The UK Biobank project number for this study was 97,089.

### Covariables and outcomes

Extensive review of established evidence was conducted to identify the factors influencing the relationship between CRP and AF in CSs [12, 16]. Various variables were extracted from the baseline assessment data of participants, covering sociodemographic factors such as age, gender, race, and educational attainment (college or university). Lifestyle factors examined included body mass index (BMI), waist circumference, smoking habits, alcohol consumption, dietary preferences, and frequency of moderate-intensity physical activity exceeding 10 min per week. Additionally, clinical aspects like CRP levels, confirmed diabetes mellitus, hypertension, history of coronary artery disease(CAD), heart failure(HF), ischemic stroke, chemotherapy, radiotherapy and the usage of medications for hypertension, diabetes, or statins were also considered.

Specific cutoff values were applied for key variables, with age limited to 65 years, BMI capped at 30 kg/m^2^, and waist circumference set at 90 cm. CRP levels were standardized at 2 mg/L, in alignment with insights from prior clinical research and the definition of residual inflammatory risk [17–19]. 

All-cause mortality and AF-related death outcomes were determined from death registry data, while new-onset AF events were classified according to ICD-10 codes (Table S1). The mean follow-up period for HES and mortality data amounted to 10.4 ± 1.5 years (IQR 9.8–11.3 years). For detailed information on each covariate, measurement techniques, and the programming codes used for covariate computation, please consult Table S1.

### Statistical analysis

The data analysis was conducted utilizing appropriate statistical methods. Normally distributed continuous variables were represented as mean ± standard deviation (SD), while non-normally distributed continuous variables were presented as median and interquartile range (IQR). Categorical variables were expressed as counts and percentages. Variances between groups were evaluated using Pearson’s chi-square test. The primary outcome of the study was the occurrence of AF, with non-AF-related death considered as a competing risk.

Participant characteristics were detailed in counts and percentages, with group variations assessed using the Pearson’s chi-square test. Initial univariate analyses were carried out, followed by the plotting of Cumulative Incidence Function (CIF) curves to visualize changes in AF incidence over time. The Fine-Gray test was employed to compare differences between groups. Subsequently, independent risk factors for AF development were explored using competing risk regression with Cox proportional hazards models. Interaction effects on both multiplicative and additive scale were analyzed through the likelihood ratio test and calculations of relative excess risk due to interaction.

A two-sided P-value of 0.05 was applied for determining statistical significance. All statistical computations were executed using R version 4.4.2 and R Studio Version 1.4.1717.

## Results

### Participant characteristics

The participants’ characteristics are provided in Table 1.

**Table 1:** Participant characteristics

	CRP ≤ 2 mg/L	CRP > 2 mg/L	*P*-value
Number, n (%)	11,826(60.1%)	7851(39.9%)	
Socio-demographics			
Age, years	59.6 ± 7.2	60.2 ± 6.9	< 0.001
Gender			
Female	7692(65.0%)	5254(66.9%)	0.007
Male	4134(35.0%)	2597(33.1%)	
Race			
White	11,546(97.6%)	7653(97.5%)	0.659
Asian	78(0.7%)	64(0.8%)	
Black	79(0.7%)	53(0.7%)	
Mixed or other	123(1.0%)	81(1%)	
College or university degree			
No	7770(65.7%)	5925(75.5%)	< 0.001
Yes	4056(34.3%)	1926(24.5%)	
Lifestyle characteristics			
BMI	26.1 ± 4.0	29.5 ± 5.4	< 0.001
Waist circumference	86.4 ± 12.4	94.7 ± 13.4	< 0.001
Number of times per week consuming processed meats			
None	1334(11.3%)	607(7.7%)	< 0.001
1 ~ 3	10,137(85.7%)	6973(88.8%)	
4+	355(3.0%)	271(3.5%)	
Number of times per day consuming fruit or vegetables			
None	415(3.5%)	378(4.8%)	< 0.001
1 ~ 2	5050(42.7%)	3484(44.4%)	
3 ~ 4	5083(43.0%)	3222(41.0%)	
5+	1278(10.8%)	767(9.8%)	
Alcohol consumption			
Daily or almost daily	2696(22.8%)	1430(18.2%)	< 0.001
Three or four times a week	2766(23.4%)	1472(18.7%)	
Once or twice a week	2968(25.1%)	1979(25.2%)	
One to three times a month	1206(10.2%)	940(12.0%)	
Special occasions only	1333(11.3%)	1236(15.7%)	
Never	857(7.2%)	794(10.1%)	
Smoke	7221(61.1%)	5129(65.3%)	< 0.001
Days per week spent doing moderate physical activity > 10 min			
None	1364(11.5%)	1344(17.1%)	< 0.001
1 ~ 2	2578(21.8%)	1687(21.5%)	
3 ~ 7	7484(66.7%)	4820(61.4%)	
Clinical characteristics			
C-reactive protein, mg/L	0.9(0.5–1.3)	3.9(2.7–6.6)	<0.001
Hypertension	3705(31.3%)	3186(40.6%)	< 0.001
Diabetes	647(5.5%)	600(7.6%)	< 0.001
Coronary artery disease	572(4.8%)	379(4.8%)	1
Heart failure	7(0.1%)	16(0.2%)	0.007
Ischemic stroke	258(2.2%)	212(2.7%)	0.022
On antihypertensive medication	2397 (20.3%)	1888(24.0%)	< 0.001
On statin	2418(20.4%)	1505(19.2%)	0.029
On diabetes medication	378(3.2%)	326(4.2%)	< 0.001
Chemotherapy	1223(10.3%)	1109(14.1%)	< 0.001
Radiotherapy	287(2.4%)	229(2.9%)	0.039

The group with CRP level > 2 mg/L accounted for 39.9% of the participants. The mean age of this group was 60.2 (± 6.9) years, while the mean age of the CRP ≤ 2 mg/L group was slightly lower at 59.6(± 7.2) years. The proportion of male participants was higher in the CRP ≤ 2 mg/L group, constituting 35.0% of the total participants (*n* = 4,134). Moreover, the CRP ≤ 2 mg/L group exhibited a higher level of educational accomplishment. The CRP > 2 mg/L group displayed more unhealthy lifestyle traits, including obesity, increased consumption of processed meats, lower intake of fruits and vegetables, lack of physical activity, and a higher prevalence of smoking. Additionally, this group had a higher incidence of comorbidities such as hypertension, diabetes mellitus, HF, and ischemic stroke, with a larger percentage of individuals taking medications for hypertension and diabetes. Moreover, a higher proportion had undergone prior radiotherapy and chemotherapy. Conversely, the CRP ≤ 2 mg/L group had higher rates of alcohol consumption and statin usage.

Over a mean follow-up period of 10.4 ± 1.5 years, 836 cases of AF were documented, resulting in an incidence rate of 4.25 per 100 individuals. Among these cases, 391 (4.98 per 100 individuals) were observed in the CRP > 2 mg/L group, while 445 (3.76 per 100 individuals) occurred in the CRP ≤ 2 mg/L group. The incidence of AF was higher in the CRP > 2 mg/L group compared to the CRP ≤ 2 mg/L group. During follow-up, there were 1,422 all-cause deaths, with AF accounting for only two cases.

### Correlates of new-onset AF in CSs

The analysis conducted to identify independent risk factors for AF in CSs utilized multivariable analyses with competing risk regression and Cox proportional hazards model, presented in Tables 2, 3 and 4. The results revealed several factors significantly correlated with the development of AF within this specific population. The multivariable analysis underscored that factors associated with AF in CSs comprised age ≥ 65 years (HR 2.20, 95% CI 1.90–2.55; *P* < 0.001), male gender (HR 1.73, 95% CI 1.46–2.05; *P* < 0.001), BMI ≥ 30 kg/m^2^ (HR 1.34, 95% CI 1.14–1.58; *P* < 0.001) and waist circumference ≥ 90 cm (HR 1.24, 95% CI 1.03–1. 50; *P* = 0.025), as shown in Tables 2, 3 and 4. Additionally, factors such as a history of CAD (HR 1.74, 95% CI 1.39–2.17; *P* < 0.001), a history of HF (HR 3.66, 95% CI 1.48–9.02; *P* = 0.005), use of antihypertensive medication (HR 1.44, 95% CI 1.19–1.75; *P* < 0.001) and previous chemotherapy (HR 1.24, 95% CI 1.01–1.53; *P* = 0.043) were also significantly associated with the occurrence of AF in the studied population as indicated in Tables 2, 3 and 4. However, engaging in moderate physical activity exceeding 10 min 1–2 days per week and a higher education level were associated with a reduced incidence of AF, with hazard ratios of 0.78 (95% CI 0.63–0.96; *P* = 0.019) and 0.84 (95% CI 0.71–0.99; *P* = 0.035), respectively (Table 4).

**Table 2:** Associations between CRP and AF in CSs in model 1

Variables	sHR	95%CI	*P*-value
Socio-demographics			
Age			
< 65, years	Reference		
≥65, years	2.33	2.03–2.67	< 0.001
Gender			
Female	Reference		
Male	2.19	1.90–2.51	< 0.001
Race			
White	Reference		
Asian	0.53	0.17–1.63	0.27
Black	0.51	0.16–1.61	0.25
Mixed or other races	1.24	0.66–2.33	0.50
College or university degree			
No	Reference		
Yes	0.77	0.65–0.90	0.001
C-reactive protein, mg/L			
≤2	Reference		
>2	1.30	1.14–1.49	< 0.001

**Table 3:** Associations between CRP and AF in CSs in model 2

Variables	sHR	95%CI	*P*-value
Socio-demographics			
Age			
< 65, years	Reference		
≥65, years	2.13	1.85–2.46	< 0.001
Gender			
Female	Reference		
Male	1.91	1.66–2.21	< 0.001
Ethnic			
White	Reference		
Asian	0.48	0.16–1.44	0.19
Black	0.47	0.15–1.46	0.19
Mixed or other races	1.29	0.69–2.42	0.43
College or university degree			
No	Reference		
Yes	0.81	0.69–0.95	0.011
Clinical characteristics			
C-reactive protein, mg/L			
≤2	Reference		
>2	1.21	1.06–1.37	0.005
Diabetes			
No	Reference		
Yes	0.99	0.69–1.42	0.97
Hypertension			
No	Reference		
Yes	1.24	1.03–1.48	0.02
Coronary artery disease			
No	Reference		
Yes	1.78	1.43–2.23	< 0.001
Heart failure			
No	Reference		
Yes	3.57	1.42–8.99	0.007
Ischemic stroke			
No	Reference		
Yes	1.29	0.92–1.80	0.14
On antihypertensive medication			
No	Reference		
Yes	1.44	1.19–1.75	< 0.001
On diabetes medication			
No	Reference		
Yes	1.03	0.66–1.61	0.89
On statin			
No	Reference		
Yes	1.02	0.86–1.21	0.80
Chemotherapy			
No	Reference		
Yes	1.24	1.01–1.53	0.04
Radiotherapy			
No	Reference		
Yes	1.05	0.69–1.58	0.83

**Table 4:** Associations between CRP and AF in CSs in model 3

Variables	sHR	95%CI	*P*-value
Socio-demographics			
Age			
< 65, years	Reference		
≥65, years	2.20	1.90–2.55	< 0.001
Gender			
Female	Reference		
Male	1.73	1.46–2.05	< 0.001
Ethnic			
White	Reference		
Asian	0.50	0.17–1.51	0.22
Black	0.46	0.15–1.43	0.18
Mixed or other races	1.27	0.67–2.41	0.47
College or university degree			
No	Reference		
Yes	0.84	0.71–0.99	0.035
Lifestyle characteristics			
BMI			
<30 kg/m^2^	Reference		
≥30 kg/m^2^	1.34	1.14–1.58	< 0.001
Waist circumference			
<90 cm	Reference		
≥90 cm	1.24	1.03–1.50	0.025
Number of times per week consuming processed meats			
None	Reference		
1 ~ 3	0.91	0.70–1.18	0.46
4+	0.83	0.54–1.27	0.40
Number of times per day consuming fruit or vegetables			
None	Reference		
1 ~ 2	0.83	0.59–1.15	0.26
3 ~ 4	0.81	0.58–1.12	0.21
5+	0.94	0.65–1.36	0.74
Alcohol consumption			
Daily or almost daily	Reference		
Three or four times a week	0.93	0.76–1.15	0.52
Once or twice a week	0.96	0.78–1.17	0.66
One to three times a month	0.96	0.74–1.25	0.77
Special occasions only	0.85	0.66–1.10	0.21
Never	0.98	0.74–1.30	0.88
Smoke			
No	Reference		
Yes	1.14	0.98–1.33	0.1
Days per week spent doing moderate physical activity > 10 min			
None	Reference		
1 ~ 2	0.80	0.63–1.00	0.05
3 ~ 7	0.87	0.72–1.05	0.15
Clinical characteristics			
C-reactive protein, mg/L			
≤2	Reference		
>2	1.14	0.99–1.31	0.076
Diabetes			
No	Reference		
Yes	0.92	0.64–1.32	0.65
Hypertension			
No	Reference		
Yes	1.17	0.97–1.40	0.10
Coronary artery disease			
No	Reference		
Yes	1.74	1.39–2.17	< 0.001
Heart failure			
No	Reference		
Yes	3.66	1.48–9.02	0.005
Ischemic stroke			
No	Reference		
Yes	1.27	0.91–1.77	0.16
On antihypertensive medication			
No	Reference		
Yes	1.44	1.19–1.75	< 0.001
On diabetes medication			
No	Reference		
Yes	0.99	0.63–1.55	0.97
On statin			
No	Reference		
Yes	1.00	0.84–1.18	0.96
Chemotherapy			
No	Reference		
Yes	1.24	1.01–1.53	0.043
Radiotherapy			
No	Reference		
Yes	1.03	0.68–1.56	0.88

Given the propensity for hypertensive medications to potentially contribute to the new-onset AF, an in-depth examination into the baseline characteristics of individuals taking such medications was undertaken (Table S2). The findings revealed that these individuals, predominantly older males with lower levels of education, exhibited habits such consuming processed meats, frequent alcohol intake, smoking, physical inactivity and higher level of CRP. Additionally, they presented common comorbidities including obesity, hypertension, diabetes mellitus, CAD, HF and ischemic stroke. Furthermore, they were more inclined to be prescribed diabetes medications and statins, indicating a pattern of interconnected health issues among them.

### Association of CRP with new-onset AF in CSs

**Fig. 2:**
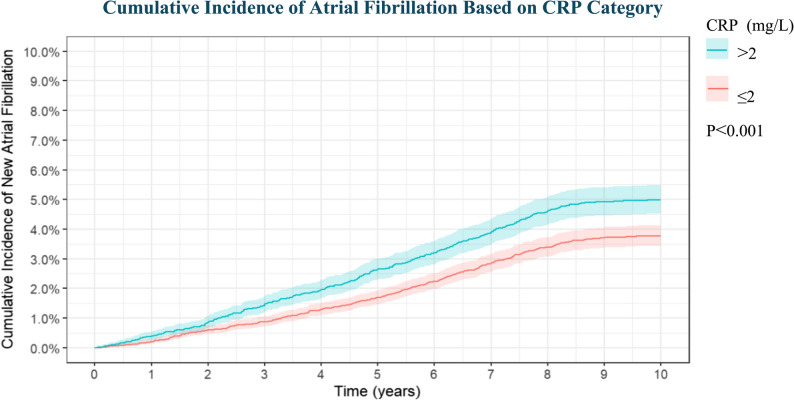
Cumulative Incidence of Atrial Fibrillation Based on CRP Category. CRP, C-reactive protein. AF, atrial fibrillation. CSs, cancer survivors. P-values were calculated between each exposure group and the cumulative incidence of new-onset atrial fibrillation using the Fine-Gray test, accounting for competing risks of non-AF-related mortality

In our investigation, a univariable competing risks model was utilized to assess to the cumulative incidence of AF in relation to CRP > 2 mg/L. The findings illustrated in Fig. 2 displayed a substantial correlation between elevated CRP levels and an heightened cumulative risk of AF. To delve deeper into the independent relationship between CRP levels and AF, a multivariable competing risk model was constructed. In Model 1 (sociodemographics only), CRP levels > 2 mg/L independently predicted incident AF (HR 1.30, 95% CI 1.14–1.49, *P* < 0.001) (Table 2). The association remained robust after additional adjustment for clinical covariates in Model 2 (HR 1.21, 95% CI 1.06–1.37; *P* = 0.005) (Table 3). Further inclusion of lifestyle factors in Model 3 attenuated the effect, yet CRP > 2 mg/L retained a borderline significant trend (HR 1.14, 95% CI 0.99–1.31, *P* = 0.076) (Table 4).

Among eight common cancer subtypes, we analyzed the relationship between CRP levels and the incidence of new-onset AF. The results showed a trend toward increased AF incidence with elevated CRP levels across most cancer types, with the exception of lymphoma. However, this association did not reach statistical significance (Table S3).

### Subgroup analyses

In the subgroup analysis documented in Table 5, a detailed exploration was conducted into the potential interaction between CRP levels > 2 mg/L and the risk of AF in various subgroups of CSs. The findings indicated that that inflammation, signaled by CRP > 2 mg/L, amplified the susceptibility to AF independently of variables such age, gender, BMI, waist circumference, smoking, diabetes or hypertension status, history of CAD and HF, the use of medications for hypertension or statins, or prior chemotherapy—without evidence of effect modification (all P for interaction > 0.05) (Table 5). Essentially, the adverse impacts of inflammation remained uniform across the described subgroups of CSs, demonstrating consistency rather than variation based on these specific attributes. Furthermore, the influence of inflammation on AF was more pronounced in CSs who had not received radiotherapy (P for interaction = 0.032), suggesting that the absence of prior radiation may amplify the arrhythmogenic impact of inflammation.

**Table 5:** Subgroup analysis of CRP levels and the risk of AF occurrence in CSs

Stratified variables			HR (95% CI)		*P* value	*P* for interaction
	Number	Percent	CRP ≤ 2 mg/L	CRP > 2 mg/L		
Overall	19,677	100	1	1.33(1.16–1.53)	< 0.001	
Age, years						0.126
<65	13,625	69.2	1	1.46(1.20–1.78)	< 0.001	
≥65	6052	30.8	1	1.18(0.98–1.42)	0.082	
Gender						0.193
Female	12,946	65.8	1	1.50(1.23–1.84)	< 0.001	
Male	6731	34.2	1	1.25(1.04–1.50)	0.017	
BMI						0.742
<30	14,800	75.2	1	1.19(1.00–1.42)	0.055	
≥30	4877	24.8	1	1.13(0.89–1.44)	0.316	
Waist circumference				0.617		
< 90 cm	10,124	51.5	1	1.16(0.90–1.50)	0.261	
≥ 90 cm	9553	48.5	1	1.07(0.91–1.27)	0.404	
Smoke						0.762
No	7327	37.2	1	1.36(1.06–1.74)	0.014	
Yes	12,350	62.8	1	1.30(1.10–1.53)	0.002	
Diabetes						0.895
No	18,430	93.7	1	1.31(1.14–1.52)	< 0.001	
Yes	1247	6.3	1	1.35(0.88–2.09)	0.17	
Hypertension						0.50
No	12,786	65.0	1	1.31(1.08–1.60)	0.007	
Yes	6891	35.0	1	1.19(0.99–1.44)	0.067	
Coronary artery disease						0.314
No	18,726	95.2	1	1.37(1.19–1.59)	< 0.001	
Yes	951	4.8	1	1.11(0.76–1.63)	0.583	
Heart failure						0.389
No	19,654	99.9	1	1.33(1.16–1.53)	< 0.001	
Yes	23	0.1	1	0.62(0.10–3.75)	0.607	
On antihypertensive medication						0.069
No	15,392	78.2	1	1.42(1.19–1.69)	< 0.001	
Yes	4285	21.8	1	1.10(0.88–1.36)	0.399	
On statin						0.768
No	15,754	80.1	1	1.33(1.13–1.57)	0.001	
Yes	3923	19.9	1	1.39(1.09–1.77)	0.008	
Chemotherapy						0.884
No	17,345	88.1	1	1.33(1.15–1.53)	< 0.001	
Yes	2332	11.9	1	1.37(0.93–2.00)	0.108	
Radiotherapy						0.03
No	19,161	97.4	1	1.37(1.19–1.57)	< 0.001	
Yes	516	2.6	1	0.51(0.21–1.23)	0.136	

## Discussion

In our comprehensive study involving 19,677 individuals with a cancer history, we conducted the first extensive systematic investigation into the relationship between CRP levels and the incidence of AF. The results revealed a notable correlation, indicating that CRP > 2 mg/L were associated with increased risk of developing AF in this population. This risk was particularly prominent among individuals who had not received radiotherapy.

Furthermore, our research highlighted the prevalence comorbidities among CSs. Those with CRP > 2 mg/L exhibited a higher likelihood of various conditions such as advanced age, obesity, smoking, lack of exercise, diabetes, hypertension, CAD, and HF, all of which are known to elevate the risk of AF [20]. Additionally, traditional risk factors like advanced age, obesity, smoking, lack of exercise, CAD, and HF were found to significantly increase the risk of AF. Crucially, multivariable adjustment demonstrates that the relationship between systemic inflammation and new-onset AF persists after accounting for these conventional determinants, underscoring inflammation as an important pathophysiological driver in the CSs. Nevertheless, incorporation of lifestyle variables attenuates the magnitude of the CRP–AF association, implying that health-behaviour pathways may partially mediate or confound the inflammatory signal. These findings highlight the need to target both biological and behavioural axes when stratifying and mitigating AF risk in CSs.

An intriguing observation from our study was the link between the use of hypertensive medications and an increased risk of AF. This association may stem from individuals on such medications having a higher predisposition to AF due to multiple risk factors.

Our study unveiled a substantial and independent association between systemic inflammation and an increased risk of AF in CSs, aligning with some, but not all, previous studies in this area.

CRP serves as a straightforward and reliable indicator of systemic inflammation. Our research illustrates that individuals with CRP >2 mg/L were more likely to present with comorbid risk factors for AF such as obesity, hypertension, diabetes mellitus, and HF[20]. Moreover, elevated CRP levels were independently associated with a higher incidence of AF, regardless of these conventional risk factors. Recent large cohort studies have highlighted a strong link between CRP and AF development [21–26], leading to its recognition as a biomarker in predicting AF in the 2024 ESC guidelines [20]. 

The evolving understanding of the relationship between inflammation and AF risk has spurred investigations into the potential benefits of anti-inflammatory interventions in AF prevention and treatment. Various studies have explored anti-inflammatory therapies targeting AF, with some showing promising outcomes [15, 27–29]. For instance, recent findings demonstrated that steroid therapy reduced the risk of postoperative AF in a canine model [30] and may aid in reducing AF recurrence after catheter ablation in the clinical setting [31]. Although some anti-inflammatory strategies, like colchicine [32, 33] and statins [34, 35], have shown effectiveness in specific contexts, our study revealed that statin administration did not decrease new-onset AF events in CSs. Additionally, recent randomized controlled trials investigating colchicine administration did not show a significant decrease in the incidence of clinically significant AF in patients undergoing major noncardiac thoracic surgery [36]. The variations in trial outcomes could be attributed to several factors including participant demographics, follow-up duration, protocol discontinuation rates, and potential differences in targeted inflammatory pathways. Despite the ongoing exploration of anti-inflammatory interventions for AF management, the latest ESC guidelines do not endorse these strategies as standard practice due to the mixed results observed in clinical trials [19]. This underscores the crucial need for further research to uncover the precise role of inflammation and evaluate the impact of anti-inflammatory interventions on AF outcomes.

In cancer patients, inflammatory markers like CRP, TNF-α, and interleukins are commonly elevated [14], reflecting an inflammatory state [20]. Our study observed that a significant percentage of CSs had CRP >2 mg/L, indicating chronic low-grade inflammation [17, 18 ]. The onset of AF in cancer settings may result from comorbid conditions, direct tumor effects, treatment complications, or shared inflammatory processes [4]. There is a notion that AF could potentially be an inflammatory complication of cancer [37]. Several systematic reviews have also highlighted the link between inflammation and AF development in cancer patients [4, 16, 38, 39], yet no study has specifically examined the correlation between CRP and new-onset AF in CSs. Our findings strongly suggest that low-grade inflammation, as indicated by CRP levels, is a key independent risk factor for AF development in CSs. This emphasizes the potential of utilizing CRP as a biomarker for monitoring CTR-CVT and implementing anti-inflammatory interventions to reduce AF risk in this vulnerable population.

The specific mechanisms through which inflammation heightens the risk of AF in CSs remain incompletely understood. However, drawing on previous research, several potential mechanisms can be hypothesized.

Cancer patients with chronic low-grade inflammation often present with traditional risk factors for AF such as obesity, hypertension, CAD and HF [39]. Treatments like anticancer drugs, radiation therapy, and surgery can initiate AF through inflammatory pathways [4]. Interestingly, early histologic studies have indicated that even in individuals with “isolated AF,” around two-thirds of patients showcase localized atrial inflammatory infiltrates akin to myocarditis [40]. The inflammatory response mediators have the potential to impact atrial electrophysiology and structural substrates, thereby increasing susceptibility to AF [41]. Inflammation can also modulate calcium homeostasis and connexins, which play a role in AF triggers and the heterogeneous conduction of the atria [41]. Moreover, the relationship between AF and inflammation is bidirectional, as AF itself can exacerbate inflammation, leading to the phenomenon known as ‘AF begets AF’ [41]. Furthermore, inflammation has been linked to expediting the aging process and fostering the clonal expansion of cells in normal tissues, including blood cells [42]. Recent research have unveiled that clonal hematopoiesis of indeterminate potential (CHIP) serves as a risk factor for both cancer [43–45] and AF [45, 46]. Continued investigation is imperative to fully uncover the potential mechanisms through which inflammation heightens the risk of AF in CSs. This deeper comprehension will facilitate the development of targeted interventions to mitigate AF risk in this specific population.

### Strengths and limitations

Our research possesses several notable strengths that enhance its effectiveness and reliability. A key advantage lies in the utilization of the extensive and well-established UK Biobank database, providing robust and detailed measurements of various exposure factors. The database’s long-term and rigorous follow-up procedures help mitigate common biases prevalent in observational studies, such as selection, recall, and information bias. Furthermore, our study considered the traditional risk factors for AF in CSs. By incorporating a comprehensive evaluation of common AF risk factors and treatment medications in our analysis, we aimed to address potential confounding variables. Notably, our findings suggest that the detrimental impact of elevated CRP levels on new-onset AF in CSs remains significant independent of baseline traditional risk factors. This indicates the potential universal applicability of CRP as a predictive biomarker for AF across a broader population of CSs.

It is essential to acknowledge the limitations of our study, despite its strengths. One significant limitation was the limited statistical power, which restricts the ability to perform robust analyses—particularly due to small sample sizes within individual cancer subtypes, the presence of variables with only one level, and the absence of detailed radiotherapy and chemotherapy regimens. Additionally, the study focused mainly on Caucasian participants, restricting the generalizability of the results to other racial groups. These limitations should be considered when interpreting our findings, highlighting the necessity for further research to bridge these knowledge gaps and ensure inclusivity across diverse populations.

## Conclusion

Our findings suggest that systemic inflammation is associated with an increased risk of AFamong CSs,. particularly in individuals without a history of radiotherapy. Further studies are needed to explore underlying mechanisms and therapeutic implications.

## Supplementary Information

Supplementary Material 1.

## Data Availability

Please visit the UK Biobank Data Showcase at https://biobank.ndph.ox.ac.uk/showcase.

## Declarations

### Permission information

The authors do hereby declare that all illustrations and figures in the manuscript are entirely original and do not require reprint permission.
