# Supplementary material for: Association between systemic inflammation and risk of atrial fibrillation in cancer survivors: a population-based cohort study using UK biobank
Source: Cardiooncology. 2025 Dec 12;12:8. doi: 10.1186/s40959-025-00414-6 (PMC12822089; doi:10.1186/s40959-025-00414-6)
Supplement: Supplementary file 1 — Supplementary Material 1. [file 40959_2025_414_MOESM1_ESM.docx]

| **Table S1.Codelists for Outcomes, Exposure, and Covariates** | | |
| --- | --- | --- |
| Codelists for outcomes, exposure, and covariates | UKB Data-Field Number | Description or ICD10 code |
| Age | n_21003 _* | This is a derived variable based on date of birth and date of attending assessment centre and refers to the age of the participant on the day they attended an Assessment Centre, truncated to whole year part. |
| Date of attending assessment centre | s_53_* | Date when a participant attended a UK Biobank assessment centre. |
| Gender | n_31_* | Sex of participant. |
| Ethnic | n_21000_* | This is an amalgam of sequential branching questions asked during the initial Assessment Centre visit as part of the touchscreen questionnaire. |
| College or university degree | n_6138_* | ACE touchscreen question "Which of the following qualifications do you have? |
| BMI | n_21001_* | Constructed from height and weight measured during the initial Assessment Centre visit |
| Waist circumference | n_48_* | Waist circumference |
| Number of times per week consuming processed meats | n_1349_* | ACE touchscreen question "How often do you eat processed meats (such as bacon, ham, sausages, meat pies, kebabs, burgers, chicken nuggets)?" |
| Number of times per day consuming fruit or vegetables | n_1289_* | ACE touchscreen question "On average how many heaped tablespoons of COOKED vegetables would you eat per DAY? (Do not include potatoes; put '0' if you do not eat any)" |
| Alcohol consumption | n_1558_* | ACE touchscreen question "About how often do you drink alcohol?" |
| Ever smoked | n_20160_* | Derived using variables "Current tobacco smoking" (Field 1239) and "Past tobacco smoking" (Field 1249). |
| Days per week spent doing moderate physical activity >10 mins | n_884_* | ACE touchscreen question "In a typical WEEK, on how many days did you do 10 minutes or more of moderate physical activities like carrying light loads, cycling at normal pace? (Do not include walking)" |
| C-reactive protein | n_30710_* | Measured by immunoturbidimetric - high sensitivity analysis on a Beckman Coulter AU5800 |
| Diabetes | n_20002_* | 1220, 1222, 1223 |
| Hypertension | n_20002_*, | 1065, 1072 |
| Coronary artery disease | n_20002_* | 1074, 1075 |
| Heart failure | n_20002_* | 1076 |
| Ischemic stroke | n_20002_* | 1081, 1082 |
| On antihypertensive medication | n_20003_* | Code for treatment. Negative codes indicate free-text entry. |
| On diabetes medication | n_20003_* | Code for treatment. Negative codes indicate free-text entry. |
| On statin | n_20003_* | Code for treatment. Negative codes indicate free-text entry. |
| Chemotherapy | s_41270_* | Z511, Z512 |
| Radiotherapy | s_41270_* | Z510 |
| Self-reported Cancer | n_20001_* | Code for cancer. If the participant was uncertain of the type of cancer they had had, then they described it to the interviewer (a trained nurse) who attempted to place it within the coding tree. If the cancer could not be located in the coding tree then the interviewer entered a free-text description of it. These free-text descriptions were subsequently examined by a doctor and, where possible, matched to entries in the coding tree. Free-text descriptions which could not be matched with very high probability have been marked as "unclassifiable". |
| Atrial fibrillation | s_41270_* | "I480", "I481", "I483", "I484", "I489" |
| Date of atrial fibrillation | s_41280_* | This field provides, for each participant, the date each ICD-10 diagnosis code was first recorded in either the primary or secondary position in the participant's hospital inpatient records. |
| Atrial fibrillation -related death | s_40001_* | I480, I481, I483, I484, I489 |
| All cause death | s_40001_* | Underlying/primary cause of death reported for participant. Note that this may not match the text value in [Field 40010](https://biobank.ndph.ox.ac.uk/showcase/field.cgi?id=40010) due to transcription errors at source. |
| Date of all cause death | s_40000_* | Acquired from central registry. |
| Date of atrial fibrillation-related death | s_40000_* | Acquired from central registry. |
| For further details, please visit the UK Biobank Data Showcase at https://biobank.ndph.ox.ac.uk/showcase/index.cgi. The UKB Data-Field numbers above can be searched in the Data Showcase to access highly detailed notes about instruments used, validation exercises (where applicable), screenshots of the survey, coding of the variable, and summary statistics of each variable. | | |

**Table S2. Participant Characteristics**

| On antihypertensive medication | | | |
| --- | --- | --- | --- |
|  | No | Yes | P-value |
| Number, n (%) | 15392(78.2%) | 4285(21.2%) |  |
| **Socio-demographics** |  |  |  |
| Age, years | 59.1 ± 7.3 | 62.4 ± 5.6 | <0.001 |
| Gender |  |  |  |
| Female | 10816(70.3%) | 2130(49.7%) | <0.001 |
| Male | 4576(29.7%) | 2155(50.3%) |  |
| Race |  |  |  |
| White | 15406(97.8%) | 4153(96.9%) | <0.001 |
| Asian | 102(0.7%) | 40(0.9%) |  |
| Black | 80(0.5%) | 52(1.2%) |  |
| Mixed or other | 164(1.1%) | 40(0.9%) |  |
| College or university degree | |  |  |
| No | 10527(68.4%) | 3168(73.9%) | <0.001 |
| Yes | 4865(31.6%) | 1117(26.1%) |  |
| **Lifestyle characteristics** | |  |  |
| BMI | 27.0 ± 4.7 | 29.1 ± 5.2 | <0.001 |
| Waist circumference | 88.0 ± 12.9 | 95.8 ± 13.6 | <0.001 |
| Number of times per week consuming processed meats | | |  |
| None | 1650(10.7%) | 291(6.8%) | <0.001 |
| 1~3 | 13292(86.4%) | 3818(89.1%) |  |
| 4+ | 450(2.9%) | 176(4.1%) |  |
| Number of times per day consuming fruit or vegetables | | |  |
| None | 649(4.2%) | 144(3.4%) | 0.021 |
| 1~2 | 6695(43.5%) | 1839(42.9%) |  |
| 3~4 | 6482(42.1%) | 1823(42.5%) |  |
| 5+ | 1566(10.2%) | 479(11.2%) |  |
| Alcohol consumption |  |  |  |
| Daily or almost daily | 3131(20.3%) | 995(23.2%) | <0.001 |
| Three or four times a week | 3327(21.6%) | 911(21.3%) |  |
| Once or twice a week | 3965(25.8%) | 982(22.9%) |  |
| One to three times a month | 1735(11.3%) | 411(9.6%) |  |
| Special occasions only | 1997(13.0%) | 572(13.3%) |  |
| Never | 1237(8.0%) | 414(9.7%) |  |
| Smoke | 9584(62.3%) | 2766(64.6%) | 0.007 |
| **Clinical characteristics** |  |  |  |
| C-reactive protein, mg/L | 1.5(0.7-3.1) | 1.8(0.9-3.7) | <0.001 |
| Hypertension | 2946(19.1%) | 3945(92.1%) | <0.001 |
| Diabetes | 578(3.8%) | 669(15.6%) | <0.001 |
| Coronary artery disease | 343(2.2%) | 608(14.2%) | <0.001 |
| Heart failure | 7 (0.05%) | 16 (0.40%) | <0.001 |
| Ischemic stroke | 275(1.8%) | 195(4.6%) | <0.001 |
| On statin | 1986(12.9%) | 1937(45.2%) | <0.001 |
| On diabetes medication | 296(1.9%) | 408(9.5%) | <0.001 |
|  |  |  |  |
|  |  |  |  |

**Table S3. Associations Between CRP and AF in Cancer Subtypes**

| Cancer subtypes | Model1 | | | | Model2 | | | Model3 | | |
| --- | --- | --- | --- | --- | --- | --- | --- | --- | --- | --- |
|  | sHR | 95%CI | P-value | | sHR | 95%CI | P-value | sHR | 95%CI | P-value |
| Lung cancer | 1.19 | 0.47-3.00 | | 0.72 | NA | NA | NA | NA | NA | NA |
| Breast cancer | 1.4 | 1.01-1.94 | | 0.04 | 1.33 | 0.95-1.84 | 0.09 | 1.23 | 0.87-1.74 | 0.24 |
| Skin cancer | 1.28 | 0.96-1.70 | | 0.09 | 1.27 | 0.95-1.69 | 0.1 | 1.11 | 0.82-1.51 | 0.49 |
| Colorectal cancer | 1.13 | 0.69-1.83 | | 0.63 | 1.11 | 0.66-1.87 | 0.69 | 1 | 0.58-1.72 | 0.99 |
| Prostate cancer | NA | NA | | NA | NA | NA | NA | NA | NA | NA |
| Cervical cancer | NA | NA | | NA | NA | NA | NA | NA | NA | NA |
| Bladder cancer | 0.69 | 0.32-1.48 | | 0.34 | NA | NA | NA | NA | NA | NA |
| Lymphoma | 1.04 | 0.56-1.91 | | 0.9 | 1.03 | 0.56-1.9 | 0.93 | 0.89 | 0.47-1.68 | 0.73 |
